# The Dynamics of SARS-CoV-2 (RT-PCR) Testing

**DOI:** 10.1155/2021/6688303

**Published:** 2021-05-22

**Authors:** Nicole Joyce, Lynsey Seim, Michael Smerina

**Affiliations:** Assistant Professor of Medicine, Hospital Internal Medicine, Mayo Clinic, Jacksonville, FL, USA

## Abstract

The COVID-19 pandemic caused by severe acute respiratory syndrome coronavirus 2 (SARS-CoV-2) is estimated to have affected 6.2 million people in the United States and 27.5 million people worldwide as of September 9, 2020. On February 2, 2020, the Secretary of the Department of Health and Human Services (HHS) determined that the public health emergency justified the development and emergency use of “in vitro diagnostics for the detection and/or diagnosis of the virus that causes COVID-19” by activating the Emergency Use Authorization (EUA) authority under section 564 of the Federal Food, Drug, and Cosmetic Act. Unfortunately, effective mitigation efforts were thwarted early in the outbreak resulting in an expansion of the initial EUA on February 29, 2020, to improve accessibility to in vitro diagnostic testing. Expectantly, the development and deployment of SARS-CoV-2 testing including RT-PCR expanded rapidly in the weeks following the EUA expansion. These newly developed and approved SARS-CoV-2 RT-PCR tests boast impressive positive and negative agreement rates nearing 100%. Despite the exceptionally high rates of agreement, caution is advised as the RT-PCR tests approved under the COVID-19 EUA are in vitro analyses developed with samples artificially doped with SARS-CoV-2 RNA. These tests therefore do not have clinically applicable sensitivity and specificity because they lack a “gold standard” for diagnosis. Here we present three challenging cases requiring cautious interpretation of the newest generation of RT-PCR molecular detection assays, highlighting the major challenges faced by providers treating patients potentially infected with SARS-CoV-2.

## 1. Introduction

In an unprecedented world of SARS-CoV-2 infection, a medley of non-FDA-approved tests has been thrust upon providers who are now challenged with their complex and potentially inconsistent interpretation. We aim to lay an intellectual framework for the rapid expansion of SARS-CoV-2 testing via EUA FDA “authorized” studies and emphasize the limitations of rapid bedside interpretation. Paramount to this topic is the often misquoted “sensitivity and specificity” of the RT-PCR detection assays, a nonexistent test property for SARS-CoV-2 due to the lack of a gold standard for verification and validation. It is important for all providers to understand these constraints and the potentially misleading test results in the clinical context of their patients. This article brings forth the potential for misinformation and misinterpretation that could lead to undesired patient outcomes. We advocate a thorough assessment of a patient's clinical picture without isolated interpretation of RT-PCR test results for SARS-CoV-2. In an effort to guide clinicians through this unparalleled time, we present three case reports to support detailed and individualized interpretations of patient presentations in the context of both positive and negative RT-PCR tests for SARS-CoV-2. We hope a deeper understanding of testing validation, and results will improve our medical communities' ability to make a true diagnosis of clinically significant COVID-19 disease or lack thereof and open up future research of more validated tests.

## 2. Case 1

The following case is of an elderly male who presented with metabolic encephalopathy and failure to thrive that was found to have persistently positive NP RT-PCR for SARS-CoV-2 over several weeks despite a lack of symptoms typically attributed to SARS-CoV-2 infection [1].

A 90-year-old male with hypertension (HTN), well-controlled type 2 diabetes mellitus (DM2), hyperlipidemia (HLD), coronary artery disease, mild Alzheimer's dementia, and chronic kidney disease (CKD) stage 3 presented to the emergency department (ED) with generalized weakness and recurrent falls for the past few days. He was diagnosed with a possible urinary tract infection owing to an otherwise negative workup including a computed tomography (CT) head and chest radiograph (CXR). He did not initially undergo a NP RT-PCR for SARS-CoV-2 on admission due to restrictions on testing at that time. He was discharged from the ED with oral antibiotics to his assisted living facility (ALF). Four days later, he returned to the ED with encephalopathy, progressive generalized weakness, and inadequate oral intake requiring admission to the inpatient medical ward. To note: the day after admission, the patient's ALF reported an infection at their facility; however, our patient did not undergo a NP RT-PCR at that time as he had no indication for testing consistent with the Centers for Disease Control and Prevention (CDC) testing algorithm [2] (note: testing criteria have since been updated). Pertinent findings on presentation included a dry oropharynx, right lower lobe crackles, ecchymosis on lower extremities, and a significant encephalopathy. Initial workup revealed a serum creatinine of 2.46 (baseline 1.8), a procalcitonin of 0.16, and a normal CXR. He was diagnosed with failure to thrive, metabolic encephalopathy, and acute kidney injury (AKI) on CKD stage 3. He was initially treated with isotonic saline for volume repletion and general supportive care. On hospital day (HD) four, the patient's metabolic encephalopathy persisted despite rehydration and resolution of his AKI warranting further investigation. Aside from a CRP of 57.4, his lab work, blood cultures, repeat procalcitonin (0.19), magnetic resonance imaging (MRI) of the brain, repeated CXR, and electroencephalogram (EEG) were unrevealing. Although the patient did not meet CDC testing criteria at that time, he underwent NP-PCR for SARS-CoV-2 on HD nine. On HD 10, NP-PCR for SARS-CoV-2 was positive and the patient was placed on modified droplet precaution while exposed staff members were instructed to self-quarantine for 14 days. A lumbar puncture (LP) was performed given the concern for encephalopathy related to COVID-19. PCR on CSF was negative for SARS-CoV-2. His encephalopathy improved by HD 20 without additional therapies; unfortunately, his discharge was delayed secondary to persistently positive SARS-CoV-2 PCR tests (Table 1). On HD 30, CRP was retested and found to be improved at 8.4. He was discharged on HD 33 after two consecutive negative RT-PCR results. During his hospital course, he remained afebrile without pulmonary symptoms, imaging findings, supplemental oxygen requirements, or other signs of infection. All blood, urine, and LP cultures remained negative, and his metabolic encephalopathy was attributed to an atypical COVID-19 infection. Modified droplet precautions were removed once he had two consecutive negative NP RT-PCR tests, and he was discharged to his ALF without further follow-up or subsequent hospitalizations [3].

## 3. Case 2

The following case is of a middle-aged female who was admitted for the treatment of a hepatohydrothorax with negative NP RT-PCR SARS-CoV-2 testing. She was readmitted hours after discharge for an empyema, with a repeat negative NP RT-PCR for SARS-CoV-2, and subsequently developed COVID-19 pneumonia. After 12 days of hospitalization, the NP RT-PCR test detected SARS-CoV-2 concurrently with the detection of SARS-CoV-2 IgG antibodies (Ab) in her serum.

A fifty-three-year-old Caucasian female with end-stage liver disease (ESLD), recurrent hepatohydrothorax, laparoscopic banding with takedown and sleeve gastrectomy, and anxiety and depressive disorder currently undergoing liver transplant evaluation initially presented to an outside hospital with a complaint of dyspnea. She was admitted with hypoxia and a large right pleural effusion. The patient developed hypotension of unknown etiology four days into admission and was transferred to the transplant intensive care unit (T-ICU) at our facility for further management. On presentation to our facility, she was afebrile with initial CXR demonstrating a moderate-large right pleural effusion with passive atelectasis in the right middle and upper lung zones and clear left lung fields. The patient had no known contact with persons with COVID-19 and had a negative NP RT-PCR SARS-CoV-2 test at the time of transfer. The patient received a pleural pigtail catheter to manage her recurrent right hepatohydrothorax with significant improvement in symptoms on subsequent imaging. She remained afebrile, normotensive, and improved to baseline; she was discharged home after 10 days of hospital care.

Within hours of returning home, the patient re-presented to the ED febrile, encephalopathic, tachycardic, and tachypneic with a maximum temperature of 38.9 degrees Celsius. Pertinent data included a leukocytosis of 12.9, creatinine 1.5 (base 0.6), lactic acid 5.1 (from 2.5 the day prior), procalcitonin 0.34 (from 0.06 ten days prior), and a repeat NP RT-PCR SARS-CoV-2 that was negative. She was admitted to the T-ICU and initiated on empiric piperacillin-tazobactam, vancomycin, and caspofungin for a suspected empyema. The patient initially responded; however, she began spiking fevers on HD eight. A CT chest 10 days after readmission was obtained and revealed a new, dense left-lower lobe consolidation with ground-glass opacities in the right upper lobe and anterior left upper lobe with a resolution of her effusion. Despite empiric antimicrobial treatment, she became progressively hypoxic.

She underwent a repeat NP RT-PCR test for SARS-CoV-2 along with serology for IgG Ab by ELISA and both resulted positively (Table 2). At that time, she was diagnosed with COVID-19 pneumonia and placed on modified droplet precautions. A bronchoscopy with bronchoalveolar lavage (BAL) was performed thirteen days after readmission; the only positive finding was a positive PCR test for SARS-CoV-2. A repeat CXR fifteen days after readmission revealed worsening bilateral interstitial and airspace opacities; she received lenzilumab for two consecutive treatments and two days later received convalescent plasma. Her COVID-19 pneumonia improved thereafter, and modified droplet precautions were removed once she had two consecutive negative NP RT-PCR tests [3]. She was recommended to wait 21 days from symptom resolution and have two negative COVID tests 24 hours apart to be relisted for liver transplant (Table 2). Interestingly, her pleural fluid culture from readmission and BAL cultures revealed no growth.

## 4. Case 3

The following case is of a middle-aged man recently recovered from severe COVID-19 pneumonia who presented with acute hypoxic respiratory failure and a positive NP RT-PCR for SARS-CoV-2.

A sixty-one-year-old Caucasian male with HTN, HLD, DM2, and end-stage renal disease on hemodialysis secondary to diabetic nephropathy presented to the ED with two days of progressively worsening cough productive of blood-tinged sputum, dyspnea, nausea, and vomiting. He had also been experiencing progressive weakness and diarrhea for the preceding 10 days. He had no known exposure to COVID-19. Upon initial exam, he was afebrile and hemodynamically stable with an oxygen saturation (SaO_2_) of 97% on room air. He was noted to have mild tachypnea and coarse crackles in the bilateral lung fields with the CXR revealing bilateral multifocal consolidations predominantly involving the mid- to lower lung zones. He rapidly deteriorated to a SaO_2_ of 71% and was placed on a 100% nonrebreather. Within 12 hours of initial presentation, the patient required mechanical ventilation and was admitted to the intensive care unit. A NP RT-PCR test for SARS-CoV-2 taken on admission returned positive prompting a diagnosis of COVID-19 pneumonia, and he was placed on modified droplet precautions.

The patient was initially treated with hydroxychloroquine and azithromycin. Despite having a prolonged hospital course complicated by an *Escherichia coli* pneumonia superinfection, his respiratory status dramatically improved after 18 days of hospital care. CXR on HD 16 showed persistence of consolidations with minimal improvement. At the time of discharge, he did not require supplemental oxygen and was discharged home.

Six days after discharge, the patient re-presented to the ED with hypoxic respiratory failure, nausea, abdominal pain, and poor oral intake. Upon exam, he was afebrile and hemodynamically stable yet tachypneic with a SaO_2_ of 84% on room air. Again, he was noted to have coarse bilateral crackles and required 15 L of supplemental oxygen to maintain a normal SaO_2_. A CXR was significant for a marked bilateral interval increase in the interstitial and airspace opacities with near-complete consolidation at the lingula, when compared to his prior admission. The radiologist noted the findings were concerning for changes of recurrent COVID-related organizing pneumonia. Initial NP RT-PCR for SARS-CoV-2 was negative upon readmission, but due to recent COVID-19 pneumonia, it was repeated the next day and found to be positive (Table 3). At this time, he was placed on modified droplet precautions given the concern for recurrent COVID-19 pneumonia.

Laboratory data were significant for a decreased ferritin of 2010 mcg/L (prior admission peak of 4157 mcg/L), a decreased CRP of 31 mg/L (prior admission peak of 193.1 mg/L), and a decreased IL-6 of 7.2 pg/mL (decreased from 67.0 pg/mL). Serum SARS-CoV-2 IgG index was obtained and was 3.58. Despite a positive NP RT-PCR for SARS-CoV-2, the patient's inflammatory markers had significantly decreased and alternate etiologies for the patient's readmission were considered. Although the patient was compliant with scheduled hemodialysis, he underwent urgent hemodialysis with ultrafiltration of 4 L with significant clinical improvement. A transthoracic echocardiogram was repeated revealing an estimated left ventricular ejection fraction (LVEF) of 40–45% compared to an LVEF of 53% just 45 days prior. At the time of discharge, the patient was breathing comfortably on room air without supplemental oxygen.

## 5. Discussion

**Figure 1:**
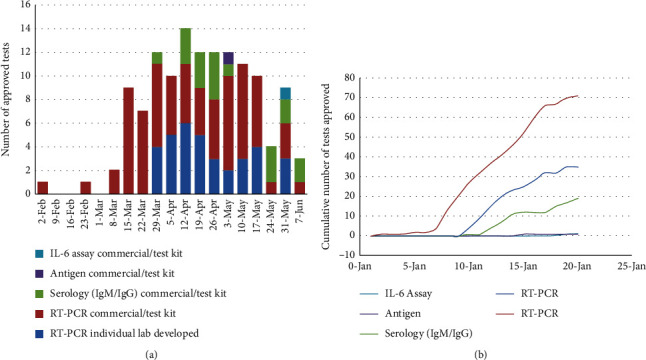
(a) Weekly EUA testing approval for SARS-CoV-2. (b) Cumulative EUA-approved testing for SARS-CoV-2.

The rapid expansion of diagnostic testing for SARS-CoV-2 in response to the EUA issued by the FDA was essential to facilitate the diagnosis and treatment of patients with COVID-19 (Figure 1(b)). As of June 2020, over 130 FDA-authorized tests to identify current and past exposure to SARS-CoV-2 exist, including molecular (RT-PCR), serology (IgM/IgG), and antigen detection [4]. Serologic testing authorized by the FDA during this unprecedented time is intended to facilitate the diagnosis of COVID-19; however, providers have been formally advised by the FDA of the limitations of current testing given the absence of a validated diagnostic tool for SARS-CoV-2 infection [5].

No available tests for SARS-CoV-2 are “FDA-approved”; rather, tests are “FDA-authorized”. The process for FDA approval is lengthy, involving scientific review, regulatory review, assessment of effectiveness and safety, evaluation of performance, review of labeling, quality control mechanisms, and other requirements [4, 6]. As of March 14, 2020, the FDA has reported the “inappropriate promotion” of serologic tests by commercial laboratories including diagnostic application and poor performance during validation trials with the National Institute of Health [7]. In addition to the limitations of testing validity, on March 13, 2020, the President issued a “Memorandum on Expanding State-Approved Diagnostic Tests” authorizing New York State along with any other state that requests the authority to do direct laboratory testing for COVID-19 without FDA validation [8].

To ensure accurate interpretation of a diagnostic test, a thorough knowledge of the methods of testing verification as well as comprehension of applications, limitations, and confounding factors that may influence results is required. Importantly, providers should familiarize themselves with the concept of agreement rates, not sensitivity and specificity. All current FDA-authorized RT-PCR testing for SARS-CoV-2 is reported in positive and negative agreement rates due to the absence of an accepted “gold standard”. Sensitivity and specificity are reserved for diagnostic tests with comparative testing to an established “gold standard” which is based on natural specimens. This does not exist for the diagnosis of COVID-19 [5, 9]; under COVID-19 EUA, in vitro analyses are with contrived samples of artificially doped SARS-CoV-2 RNA. “Agreement rates” that approach 100% may be misleading to the public and providers alike, especially when reported out of context [8, 10–12, 13]. LabCorp has reported their COVID-19 RT-PCR test as having a positive percent agreement of 100% (95% CI: 91.24%–100%) and a negative percent agreement of 100% (95% CI: 92.87%–100%); Quest Diagnostics has reported a positive and negative percent agreement of 100% (95% CI: 88.7–100%) along with a specificity of 100% (72/72, 95% CI: 95–100%), to name a few [14, 15].

Deepening the above concerns surrounding the rapid development and deployment of testing under an EUA, providers are faced with additional uncertainties surrounding testing for SARS-CoV-2. The three cases above highlight some of these uncertainties and limitations in testing and are meant to encourage thorough interpretation of all clinical and objective data without using isolated results for diagnosis and decision-making.

False-positive RT-PCR results can lead to unnecessary testing or isolation, and individual and community panic. These results are due to replication-incompetent SARS-CoV-2 on PCR testing, as demonstrated in cases one and three by persistent positive results after clinical resolution of COVID-19-related disease. This replication can persist up to 12 weeks postinfection [16]. Furthermore, the CDC updated their guidance on de-escalation of isolation precautions for patients with COVID-19 based on data showing the lack of replication-competent virus after 20 days of onset of disease. False positives suggesting true infection can also develop as a result of viral load kinetics, technical error, sample contamination, detection of an alternative microorganism, amplification errors, and separation techniques [17]. In addition, FDA recommends 100 bases for nucleic acid-based molecular diagnostic testing; however, DNA probes used in RT-PCR kits for SARS-CoV-2 are 25 bases long, which leads to increased false-positive rates [17–19].

The second case acknowledges the existence of false-negative results. These are influenced by personnel-specific procedures including standard laboratory techniques, specimen collection including location and technique, and transport and handling. Increased false negatives are also related to the timing of testing and viral shedding, and variability between prime and probe target regions in the SARS genome in the setting of mutations [20].

Solely relying on RT-PCR SARS-CoV-2 testing for diagnosis in the above three cases could have led to increased invasive testing, increased care cost, misdiagnoses, delay in diagnosis, inappropriate or delayed quarantining, superspreading, and ultimately patient and societal harm.

## 6. Conclusion

The patient presented in Case 1 above highlights the existence of persistent asymptomatic shedding as well as having a low threshold for diagnosing atypical presentations of COVID-19 infection, including encephalopathy and failure to thrive in the elderly. As a resident of an assisted living facility, a failure to initially identify his COVID-19 infection could have resulted in a significant spread to other community members and staff. We recommend maintaining a high index of suspicion for high-risk populations given the “superspreading” potential, and the need to test patients even if they lack the typical respiratory syndrome [21].

The patient presented in Case 2 above highlights the false reassurance providers may have when faced with a negative NP RT-PCR for SARS-CoV-2. The patient's clinical syndrome was consistent with COVID-19 pneumonia despite initial negative testing; she was subsequently found to have a positive NP RT-PCR along with IgG antibodies simultaneously. According to the CDC, it can take 1–3 weeks to develop antibodies to SARS-CoV-2 after becoming infected but can vary by individual [22]. This timeline would imply a false-negative SARS-CoV-2 PCR on admission. Due to the overconfidence in the sensitivity of RT-PCR for SARS-CoV-2, the patient's diagnosis and care was delayed and there was an increased risk of exposure to others. NP RT-PCR testing should not be considered in isolation but in the context of patient symptoms and laboratory and radiographic findings.

The patient presented in Case 3 above highlights the importance of expanding a differential diagnosis in the face of a positive SARS-CoV-2 PCR. Although recrudescence of COVID-19 is reported, alternate etiologies of the patient's acute hypoxic respiratory failure were considered. Cardiogenic pulmonary edema secondary to acute systolic heart failure as a consequence of his prior COVID-19 infection was ultimately diagnosed. The positive NP RT-PCR for SARS-CoV-2 at readmission was considered persistent shedding of replication-incompetent viral fragments rather than a primary recurrent pulmonary COVID-19 infectious etiology, or a false positive in clinical terms. Additionally, the CDC reports “no confirmed reports to date of a person being reinfected with COVID-19 within 3 months of initial infection” [16].

We strongly advise providers to cautiously interpret the results of SARS-CoV-2 testing in the clinical context of the patients they treat. As mentioned previously, providers should not equate the 100% positive and negative agreement rates to sensitivity and specificity of RT-PCR SARS-CoV-2 testing. Since tests were developed under an EUA, no official sensitivity or specificity data are available to date. The case examples above highlight common situations of false-positive and false-negative cases seen in clinical practice and remind providers not to rely solely on testing. The accurate diagnosis of COVID-19 infection should combine the clinical presentation and supporting findings along with an accurate interpretation of testing, taking into account the aforementioned details above.

## Figures and Tables

**Table 1:** SARS-CoV-2 PCR test results and IgG index based on hospital day, Case 1.

Case 1		
Hospital day	NP swab result	IgG index
1		
2		
3		
4		
5		
6		
7		
8		
9	Positive	
10		
11		
12		
13	Positive	
14		
15	Positive	
16		
17	Positive	
18		
19	Positive	
20		
21	Positive	
22		
23	Positive	
24		
25	Positive	
26		
27	Negative	
28	Positive	
29		
30	Negative	
31	Negative	

**Table 2:** SARS-CoV-2 PCR test results and IgG index based on hospital day, Case 2.

Case 2		
Hospital day	NP swab result	IgG index
1	Negative^*∗*^	
2		
3		
4		
5		
6		
7		
8		
9		
10		
11		
12	Positive^*∗*^	2.83
13	Positive^*∗*^	
14		
15		
16		
17		
18		
19		
20	Positive	
21		
22	Positive	
23		
24		
25	Negative	
26	Positive	
27	Negative	
28	Negative	
29		
30	Negative	

^*∗*^Symptoms of COVID-19 present, defined by CDC as fever, chills, cough, shortness of breath, fatigue, muscle aches, body aches, headache, new loss of taste or smell, sore throat, congestion or runny rose, nausea, vomiting, and diarrhea (“this list does not include all possible symptoms”) https://www.cdc.gov/coronavirus/2019-ncov/symptoms-testing/symptoms.html; https://www.cdc.gov/coronavirus/2019-nCoV/hcp/clinical-criteria.html.

**Table 3:** SARS-CoV-2 PCR test results and IgG index based on hospital day, Case 3.

Case 3					
Hospital day	NP swab result	IgG index	Hospital day	NP swab result	IgG index
1	Positive^*∗*^		1	Negative^*∗*^	
2			2	Positive	3.58
3			3		2.95
4			4	Negative	
5			5	Negative	
6			6		
7	Positive^†^		7		
8			8		
9					
10					
11					
12					
13					
14					
15					
16					
17					
18					
19					
20	Positive				
21					
22					
23					
24	Negative				
25	Positive				
26					
27					
28	Positive				
29					
30	Negative				
31	Positive				
32					
33					
34	Positive				
35	Negative				
36					
37	Negative				

^*∗*^Symptoms of COVID-19 present, defined by CDC as fever, chills, cough, shortness of breath, fatigue, muscle aches, body aches, headache, new loss of taste or smell, sore throat, congestion or runny rose, nausea, vomiting, and diarrhea (“this list does not include all possible symptoms”) https://www.cdc.gov/coronavirus/2019-ncov/symptoms-testing/symptoms.html; https://www.cdc.gov/coronavirus/2019-nCoV/hcp/clinical-criteria.html. ^†^Patient intubated at the time of testing, from suspected SARS-CoV-2-related disease.
